# A Case of Sterile Osteomyelitis: Chronic Recurrent Multifocal Osteomyelitis (CRMO)

**DOI:** 10.7759/cureus.26370

**Published:** 2022-06-27

**Authors:** Akshay Waghmode, Nikhith Soman, Aryan Gupta

**Affiliations:** 1 Radiodiagnosis, Dr. D. Y. Patil Medical College, Hospital & Research Centre, Pune, IND; 2 Medical Intern, Dr. D. Y. Patil Medical College, Hospital & Research Centre, Pune, IND

**Keywords:** bone disease, bone pain, mri, chronic non-bacterial osteomyelitis, chronic recurrent multifocal osteomyelitis, nonbacterial osteomyelitis, chronic recurrent osteomyelitis

## Abstract

Chronic recurrent multifocal osteomyelitis (CRMO) is a rarely reported autoimmune inflammatory condition affecting children and young adults. The typical complaint is recurrent severe bone pain at multiple sites; often imaging suggests features of osteomyelitis or malignancy. However, the biopsy is always negative for any growth in culture and histopathology detects only inflammatory cells. This is a diagnosis of exclusion with various diagnostic criteria. We had a 20-year-old male presenting with recurring pain and swelling in the right hand and left foot for more than seven months. Imaging revealed bone marrow edema with the expansion of bone and sclerosis involving the third metacarpal of the right hand and first metatarsal of the left foot. Acute phase reactants were mildly raised with biopsy showing inflammatory cells. However, the cultures grown from the lesion were sterile. After comparing it with the various diagnostic criteria, a diagnosis of CRMO was made.

## Introduction

Chronic recurrent multifocal osteomyelitis (CRMO) is an auto-inflammatory condition affecting multiple bones and often presents with features of osteomyelitis. It is also known as chronic nonbacterial osteomyelitis [1]. It is a very rare condition and is reported in less than 5% of all cases diagnosed as osteomyelitis. It commonly affects children and adolescents with female predominance [2]. They present with recurring severe bone pain without any fever. Even though the disease was first described in 1972 by Giedion [3], it is relatively rarely reported largely due to under-diagnosis. The diagnosis of this condition is mostly made by exclusion when no infective or neoplastic cause can be found [4,5].

## Case presentation

A 20-year-old male presented with recurrent pain and swelling in both upper and lower limbs for more than seven months, predominantly involving the right hand and left foot. He had sought treatment in multiple places and was screened for inflammatory arthritis which was negative, the initial radiographs showed no significant findings. He was given supportive treatment with limited relief. There were associated alternating episodes of remission and recurrence. There was no history of trauma, fever, weakness in limbs or any loss of sensation. On clinical examination, there was tenderness and swelling with severe restriction of movement at the third metacarpo-phalangeal joint in the right hand and first metatarso-phalangeal joint in the left foot. There was no history of any comorbidity of diabetes mellitus, immunosuppression, obesity, addictions, or any dermatological conditions. There was no significant family history of autoimmune disease.

**Figure 1:**
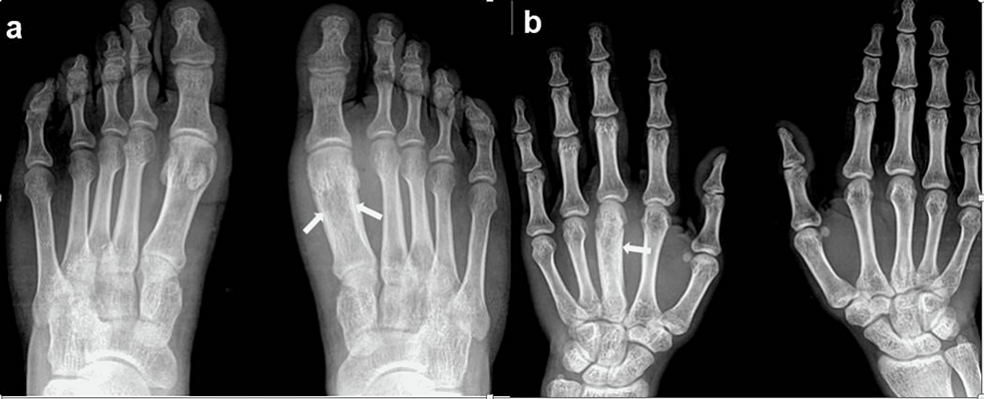
Chronic recurrent multifocal osteomyelitis (CRMO) in a 20-year-old male presented with recurrent right hand and left foot pain. Findings/technique- (a) Dorso-plantar radiograph of both feet showing widening of bone with subtle thickening of cortex and sclerosis involving the distal epiphysis, metaphysis, and diaphysis of the first metatarsal bone of the left foot. (b) Postero-anterior radiographs of both hands show similar features involving the shaft and distal end of the right third metacarpal with comparatively more evident sclerosis. The findings are more evident when compared to the contralateral normal side.

Due to the recent aggravation of symptoms, he came to a tertiary care center for detailed investigation. On admission, radiographs of all the limbs were done and there were significant findings detected in the right hand and left foot. The head and shaft of the third metacarpal appeared widened with cortical thickening and surrounding soft tissue edema (Figure 1a). Similar findings were detected on the first metatarsal of the right foot showing diaphyseal widening with areas of sclerosis and cortical thickening (Figure 1b).

In laboratory studies, erythrocyte sedimentation rate (ESR) and c-reactive protein (CRP) were mildly elevated. The total leukocyte count, rheumatoid arthritis (RA) factors, HLA-B27 antigen and antinuclear antibody (ANA) profile were all negative. In suspicion of infection, urine and blood cultures were obtained which were sterile.

**Figure 2:**
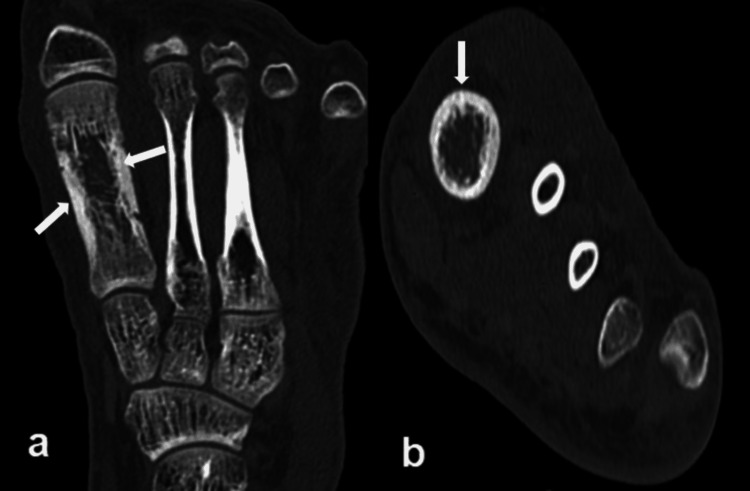
Chronic recurrent multifocal osteomyelitis (CRMO) in a 20-year-old male presented with recurrent left foot pain and swelling. Findings/technique- (a) coronal and (b) axial reformatted plain computed tomography (CT) image of the left foot showing widening of the bone, marked cortical thickening and hyperostosis due to chronic periosteal reaction in the first metatarsal bone.

CT scan of the left foot was done due to aggravated symptoms; it revealed similar findings in the first metatarsal bone with the widening of distal bone with thickening of the cortex (Figure 2).

**Figure 3:**
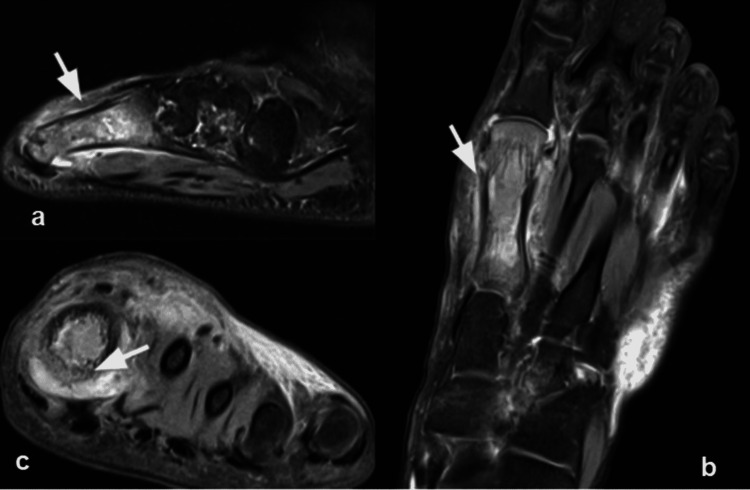
Chronic recurrent multifocal osteomyelitis (CRMO) in a 20-year-old male presented with recurrent left foot pain and swelling. Findings/technique- Non-contrast MRI (3T) of left foot with PDFS sequences in (a) sagittal, (b) coronal and (c) axial sections showing hyperintense marrow signal involving first metatarsal bone with significant cortical thickening and associated adjacent soft tissue edema.

MRI screening of the whole body was done considering the multifocal presentation of the disease; however, findings were detected only in the right hand and left foot corresponding to the radiographs. MRI of the left foot showed altered abnormal signals within the shaft of the first metatarsal with the widening of the bone and cortical thickening without break in bony cortices. The moderate hyperintense signal was seen surrounding the bone which was extending in the adjacent soft tissues and lumbricals. The first metatarso-phalangeal joint was distended with fluid and both the collateral ligaments were displaced. A few subchondral cysts were noted in the calcaneum, fourth, and fifth proximal metatarsals with surrounding marrow edema. Surrounding diffuse subcutaneous edema was also seen (Figure 3).

**Figure 4:**
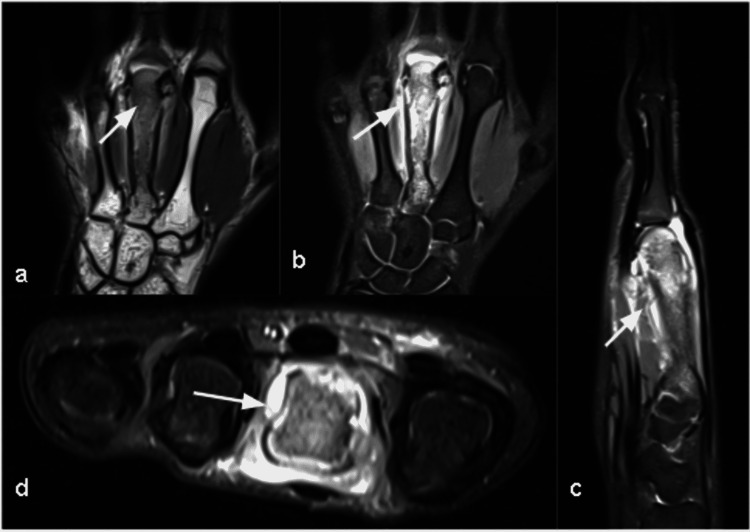
Chronic recurrent multifocal osteomyelitis (CRMO) in a 20-year-old male presented with recurrent right hand pain and swelling. Findings/technique- Non-contrast MRI(3T) of right hand on T1 weighted sequences in (a) coronal and PDFS sequences in (b) coronal, (c) sagittal and (d) axial plains showing hyperintense marrow signal involving third metacarpal with cortical thickening and widening of the bone. There is surrounding soft tissue inflammation and periosteal reaction.

MRI of the right hand showed similar findings of abnormal marrow signal within the entire third metacarpal with widening of the bone and cortical thickening. The moderate soft tissue inflammation was also seen surrounding the bone. The hyperintense signal is extending along the volar aspect and reaching up to the subcutaneous plane. The third metacarpo-phalangeal joint and both the collateral ligaments were distended with fluid with a further extension of fluid seen in the proximal one-third of the proximal phalanx of the third finger. Patchy bone marrow edema was also seen in the hamate bone (Figure 4).

After reviewing all the radiological imaging findings a provisional diagnosis of multifocal osteomyelitis was given. Another possibility of CRMO was suggested by ruling out all the infective causes. Hence, before starting any antibiotic therapy, a bone biopsy was done to rule out malignancy and infection. The histopathology studies showed inflammatory cells. The samples showed no positive growth on cultures and a test for acid-fast bacillus (AFB) was negative. The initial diagnosis of an infective or neoplastic cause was therefore ruled out. The patient was also not responding to antibiotics, hence other causes like autoimmune inflammatory conditions were considered.

Considering the age group, multifocal nature and chronicity of the disease, the diagnosis of CRMO was likely. The findings of radiological imaging also supported the diagnosis of a multifocal type of osteomyelitis with extensive marrow edema, hyperostosis, and sclerosis. The bone biopsy and microbiology were negative, which confirmed the possibility of CRMO by exclusion.

The patient was then treated with NSAIDs and oral corticosteroids. There was an immediate response and the patient was sent home with tapering doses. However, he was told to come for a follow-up for serial radiographic comparison for the progression of sclerosis or any pathological fractures.

## Discussion

Chronic recurrent multifocal osteomyelitis (CRMO) is an auto-inflammatory condition affecting multiple bones and often presenting with features of osteomyelitis. It predominantly affects metaphyseal regions of long bones, clavicles, and vertebral bodies. It is three times more common in the tubular bones of long bones as compared to other sites. The involvement of the clavicle is viewed as a typical finding since this is a rare site for infective osteomyelitis [5]. Laboratory studies are mostly inconclusive and might show mildly elevated acute phase reactants (ESR, CRP, tumour necrosis factor α [TNFα]). Cultures from blood and bone for bacterial growth are always negative [1]. The most sensitive modality for diagnosis is MRI, with the screening of the whole body for multifocal lesions [5]. In our case, there was a similar presentation in which a 20-year-old complained of recurring pain and swelling in his right hand and left foot for more than seven months. There were raised acute phase reactants with the rest of the tests being negative.

The etiology of the disease is not very clear and is considered to be an autoimmune inflammatory condition. There are strong associations with many autoimmune inflammatory dermatologic conditions like palmar-plantar pustulosis (PPP), psoriasis vulgaris, also with some inflammatory bowel disorders like Crohn’s disease, ulcerative colitis, and celiac disease. There are many rarer associations with other inflammatory conditions also noted [1]. Our patient did not have any of these related conditions. There was also no family history of any autoimmune conditions.

Few genetic associations have been established, especially in its syndromic forms. The most well known association is that of Majeed syndrome, a triad of CRMO, congenital dyserythropoietic anemia, and inflammatory dermatosis. It is a rare autosomal recessive disorder with distinct mutations in the LPIN2 gene; other mutations have also been described [6]. Another syndromic association is the deficiency of interleukin-1 receptor antagonist (DIRA), which is a fatal condition seen in newborns presenting with pustular psoriasis, non-bacterial osteomyelitis and systemic inflammation. It is associated with IL1RN mutations [7].

The radiographic findings are not very classic. However, it initially presents a metaphyseal osteolytic lesion or lytic lesions with sclerotic margins. As the chronicity of the disease increases there will be more cortical thickening and sclerosis [5,8]. The typical MRI findings are bone marrow oedema predominantly seen involving the epiphysis and metaphysis. However, it is only seen in the active phase of the disease and showing the hyperintense signal on T2 weighted images. As the inflammation subsides, sclerosis sets in with MRI showing a significant decrease in bone marrow oedema. This can be associated with periostitis and soft-tissue oedema. However, the abscess formation or fistulous tract favors infective osteomyelitis and rules out the possibility of CRMO [9]. In our patient, MRI showed classic features of active disease like bone expansion, bone marrow oedema and sclerosis involving two-thirds of the distal bone with surrounding soft tissue inflammation.

There are multiple criteria suggested for diagnosis; we followed the one suggested by Jansson et al. in 2007, as shown in Table 1 [10]. Our case met three major criteria of being sterile multifocal lesions affecting two different bones with radiological findings of osteolysis and sclerosis. The patient also had four minor criteria of symptoms lasting more than six months, normal blood count with elevated acute phase reactants and hyperostosis. Similar diagnostic criteria were also developed by Roderick et al. in 2016, known as the Bristol diagnostic criteria for CRMO [11].

**Table 1:** Clinical criteria for the diagnosis of chronic recurrent multifocal osteomyelitis (CRMO) CRP: c-reactive protein, ESR: erythrocyte sedimentation rate, PPP: palmar-plantar pustulosis

Clinical criteria for the diagnosis of CRMO. CRMO is diagnosed by two major or one major and three minor criteria.	
Major criteria	Minor criteria
Lytic/sclerotic bone lesion on imaging.	Normal blood counts with a good health condition.
Multifocal bone lesions.	Elevated CRP and ESR (Mild to moderate).
Palmo-plantar pustulosis or psoriasis.	Symptoms more than 6 months.
No growth on biopsy culture, histopathology showing inflammatory cells with changes of fibrosis.	Hyperostosis.
	Accompanying other autoimmune conditions (other than PPP or psoriasis).
	Familial history of autoimmune conditions or CRMO.
*Adapted from Jansson et al. Classification of non-bacterial osteitis [10]	

The closest differential diagnosis of infective osteomyelitis was ruled out as the cultures were sterile. On imagining infective osteomyelitis would have shown findings like fluid collection, abscess formation, fistulous tract or bony sequestrum.

The other possibilities of Langerhans histiocytosis and malignancy like Ewing sarcoma were also considered, but histopathological reports were not significant [12].

The treatment of choice is non-steroidal anti-inflammatory drugs (NSAIDs) and the majority of the patients respond well. In some refractory cases, a good outcome is seen with corticosteroids. However, patients in the long term have recurrent episodes. Currently, newer therapies are being tried with immunomodulators (anti-TNFα) [12].

## Conclusions

CRMO is a relatively unknown entity leading to under-reporting and misdiagnosis. It mainly affects the younger population and diagnosis is mainly by excluding other differentials. MRI is the most sensitive modality; whole-body screening is suggested to look for foci in other bones. The broader and well-defined diagnostic criteria have made diagnosis easier. Patients in some cases undergo years of unnecessary treatment and multiple bone biopsies. This case highlights the significance of awareness in clinicians and radiologists to consider this as a differential diagnosis in chronic recurrent cases of suspected osteomyelitis.
